# Estimating Lifetime Dental Care Expenditure in South Korea: An Abridged Life Table Approach

**DOI:** 10.3390/ijerph17093308

**Published:** 2020-05-09

**Authors:** Minsung Sohn, Xianhua Che, Sungwon Lim, Hee-Jung Park

**Affiliations:** 1 Department of Health and Care Administration, The Cyber University of Korea, 106 Bukchon-ro, Jongno-gu, Seoul 03051, Korea; minsinge@cuk.edu; 2 Department of Health Policy Research, Daejeon Public Health Policy Institute, 282 Munhwa-ro, Jung-gu, Daejeon 35015, Korea; chexianhua719@gmail.com; 3 School of Nursing, University of Washington, Health Sciences Building, Room T-507, 1959 NE Pacific Street, Seattle, WA 98195, USA; staroot1@uw.edu; 4 Department of Dental Hygiene, College of Health Science, Kangwon National University, 346 Samcheok-si, Gangwon-do 25945, Korea

**Keywords:** aging, dental expenditure, life table, life expectancy

## Abstract

The aim of this study was to measure the magnitude and distribution of a Korean’s lifetime dental expenses depending on age and sex, by constructing a hypothetical lifetime and life table of survival. Additionally, we estimated the difference in life expectancy between men and women and its impact on dental expenses. We used the 2015 Korea Health Panel Survey to calculate the total dental expenditure, including expenses paid directly by patients and those paid by insurers. We generated survival profiles to simulate dental expenses during a typical lifetime (from birth to age 95) using the abridged life table (five-year intervals for age groups) in 2015 from the South Korean Statistical Information Service. We independently calculated the remaining dental expenses for survivors of all ages. The results showed that an estimate of average lifetime dental expenditure was $31,851 per capita: $31,587 for men and $32,318 for women. Nearly 33% of the average per capita lifetime dental expenditure was attributable to the longer life expectancy of women, with no statistically significant difference in lifetime dental expenditure between men and women. Many survivors incurred 70% of their lifetime dental expenses before age 65. The results highlighted the need for policymakers to address spending on age-specific dental care owing to extended life expectancy, given the disproportionate share of healthcare resources supporting the elderly.

## 1. Introduction

Countries worldwide face the challenge of a rapidly aging population and increasing life expectancy [1]. The global number of people aged 60 years or older was estimated to be 605 million in the year 2000 and is projected to grow to nearly 2 billion by 2050 [2]. Kontis et al. have projected that life expectancy will increase continuously in developed countries, including those in the Americas, Australasia, central Europe, western Europe, and the Asia-Pacific [3]. In accordance with the worldwide aging phenomenon, South Korea has become one of the most rapidly aging nations [4,5]. The proportion of the population aged 65 years and above increased from 7% in 1999 to 11.8% in 2012, and 14% in 2017; it is expected to increase to 20.8% by 2026 [4]. According to the South Korean Statistical Information Service, the elderly population would exceed 10 million by 2025 and increase to 18.27 million by 2067 [6]. This phenomenon could become a major driver in increasing healthcare expenditures, because the elderly are at high risk for spending from chronic diseases (e.g., hypertension, diabetes, Alzheimer’s disease, and chronic lower respiratory diseases) [5,7]. 

Dental care is one of the main causes for medical expenditure. As dental diseases (e.g., tooth loss, periodontal diseases, untreated dental caries, hypo-salivation, and oral cancers) are becoming increasingly common in the current aging society, the oral health of the elderly population is of global concern [8,9,10]. In the Organization for Economic Co-operation and Development (OECD) countries, oral disease treatment accounts for an average 5% of total health expenditure [11]. Listl et al. estimated that a total of $29.8 billion was spent worldwide annually, to cover the costs of direct treatment associated with general oral conditions. This figure represents 4.6% of health expenditures worldwide [12]. Additionally, the EU reported an annual expenditure of approximately EUR 79 billion on oral care (average annual expenditure 2008–2012); if this trend continues, the expenditure may rise to 93 billion in 2020 [13]. The proportion of out-of-pocket payments in South Korea was 84%, while that for the OECD countries averages 55%; in fact, Korea’s proportion was about 3.5 times higher than that of Japan (24%) and doubled that of the United States (42%) [11].

An annual national report indicated that dental diseases such as gingivitis, periodontal diseases, and dental caries ranked among the top 10 diseases, in terms of the National Health Insurance Corporation’s (NHIC) outpatient claims over the years [14]. Korean national statistics indicated a rapid increase in the average annual spending on dental care among the elderly. Among those in the age group of 60 years or older, in particular, the proportion of dental medical expenses increased from 31.6% in 2011 to 39.9% in 2017. Dental care accounts for 23–27% of the total healthcare expenditures paid by the National Health Insurance Service (NHIS) of Korea [14]. If the rise in public dental health spending, due to low birth rates and demographic changes, is not checked, it can have a significant impact on the national finances and economy [3].

Previous studies indicated that oral diseases are chronic and cumulative, and their incidence increases with age [15,16,17]. Age could be one of the reasons that adults have higher dental expenditures than young adults. However, expenditures on dental treatments may vary by gender [18,19,20,21]. According to Wen et al., women are more likely to seek dental treatment for prevention and cure, owing to their higher knowledge and perception of oral health [20]. Lee et al. also stated that women would be more sensitive to the early stages of an oral disease than men [19]. Women, therefore, tend to seek dental care more often, resulting in less morbidity compared to that of men. Consequently, dental care spending is much higher among women than among men. However, there have been controversial differences in methodology and estimation over age–sex attributable to dental expenditures in the past because previous studies did not consider each individual’s life expectancy. Moreover, studies did not reflect the distribution of these expenses over the major phases of an individual’s lifetime, while keeping all factors other than age constant. Alemayehu and Warner focused on total medical expenditures from birth in Michigan and found that the average member of a birth cohort spent $316,579 over the course of his or her life [22]. Basu et al. also evaluated gender-based differences in hypertension-attributable to lifetime expenditures, which reported that the estimated lifetime expenditure of an average 20-year-old was $188,300 for hypertensive men and $254,910 for hypertensive women [23]. 

The results of these previous studies may be useful for policymakers to efficiently make plans and allocate resources to meet current and future needs. To the best of our knowledge, however, no previous study has estimated the lifetime dental care expenses, incorporating the survival experience of the population starting at age 0, for people who use dental care services. Therefore, it is necessary to provide information on the amount of resources required, to decision-makers in public health policies, to solve the problem of the rising dental costs of the elderly in Korea. This study used a lifetime perspective approach to construct a hypothetical individual, whose probability of being alive at each age came from a current life table, while the age–sex-specific dental expenditures were derived from cross-sectional data on age- and sex-specific spending in a single year. 

The aim of this study was to estimate the magnitude and distribution of age–sex-specific lifetime dental expenses and survival at birth by life table analysis of cross-sectional data, to construct a hypothetical lifetime. Additionally, this study measured the difference in dental expenses between men and women in a lifetime, considering their life expectancies.

## 2. Methods

### 2.1. Study Design and Data Resource 

Since 2008, the Korea National Health Insurance Service and the Korea Institute for Health and Social Affairs have conducted a national survey of households, called the Korea Health Panel Survey (KHPS). The KHPS was designed to assess national healthcare utilization and expenditure, health status, and behavior and was carried out by computer-assisted personal interviewing. A two-stage, stratified, cluster extraction method, with probability proportionality, was used for the survey sample selection. The study surveyed 18,130 people in 2015, of whom 3370 respondents had visited the dentist at least once in the previous 12 months. Respondents were required to visit the dentist and provided information about the treatment they received and the expenses incurred. The analysis of the patient-to-expense mapping was excluded. The KHPS recorded information at a maximum of two treatments per dental visit and expenses incurred for treatment, making it impossible to determine expenses for each treatment. Therefore, this study included treatment expenses for orthodontic or aesthetic purposes, in addition to oral and maxillofacial surgery costs, and direct treatment costs, for prevention and treatment.

**Figure 1:**
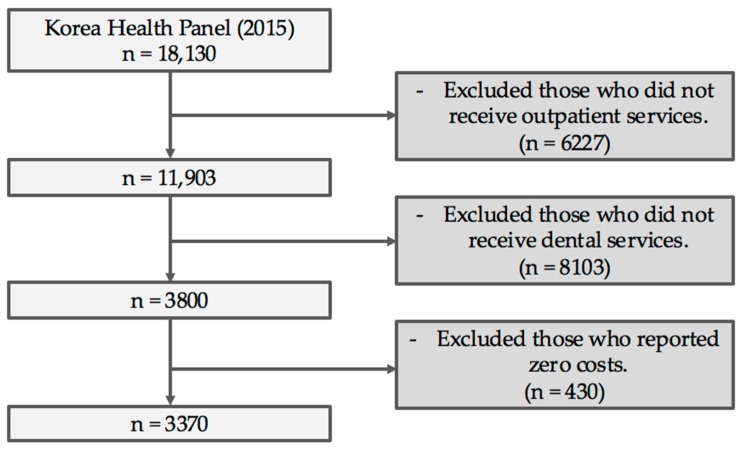
Flow of sample selection in this study.

The total dental expenses for outpatient services included those incurred by the insurer and the patients themselves. If an individual visited the dentist several times annually, the cumulative expenses were recorded. Emergency dental care cases, which were minimal, constituting only 1% of total respondents, were excluded. Cases where respondents did not know, or did not respond to the survey question on, treatment expenses were also excluded. Figure 1 describes the method used to arrive at the final sample size.

### 2.2. Analysis 

The abridged life table (five-year intervals for the age group) in 2015 from the South Korean Statistical Information Service (KOSIS) helped generate survival profiles to estimate lifetime dental expenses from birth to death (age 95) [24]. A life table is an important social indicator that identifies the structure of death for the meeting population, the life expectancy, probability of survival, and the probability of death, indicating the death status of a social population [8,25]. The current life expectancy, which is the main result of the assumption that the current age-specific death level will remain constant, and the future life expectancy are estimated. A period life table starts with the “birth” of 100,000 hypothetical persons to whom the current age-specific death rates are applied. The life table shows the mortality experience of a hypothetical group of infants born at the same time and subject throughout their lifetime to the age-specific mortality rates observed in the current period. All expenses considered were converted to dollars as per its value in 2015, accounting for the medical inflation rate.

### 2.3. Estimation of Lifetime Dental Expenditure 

A single year’s per capita dental care expenditure data and the mortality experience of a population, differentiated by age and sex, were used to generate profiles of dental care expenditure from birth to death [22]. By determining the average expenditure at each age, for each sex, and decedents and survivors, an estimated lifetime distribution of dental care expenditures was estimated. The data were converted from cross-sectional, age-specific expenditures, assuming constant income level, cost of medical and healthcare technology and healthcare services, the incidence of disease, price, and the natural history of diseases, to obtain an estimate of the per capita lifetime dental expenditure.

The average life table at birth defines per capita lifetime dental expenditure of a person as (*LE_b,a_*), at a certain age (a) after birth (b) estimating the expenditures remaining after. Although death may occur before age_a_, for some of the 100,000 cohorts, the calculation is made using 100,000 as the denominator. The per capita lifetime dental expenditure of a person (*LE_b,a_*) from birth (b) to a certain age (a) is calculated by dividing the total expenditure incurred by the cohort surviving at age_a_ by the birth cohort size (i.e., 100,000). The following equation gives the per capita lifetime dental expenditure of a person (*LE_b, a_*) from birth (b) to a certain age (a).

$$LE_{b,a}=\overset{95+}{\underset{x=a}{\sum }}CxLx/100,000$$

where, *Lχ* refers to the person-years lived by the cohort in the age interval (*χ*, *χ* + 1), and *Cχ* refers to per capita expenditure at age *χ*(*χ* = 0, 1, 2, …, 95+). The per capita lifetime expenditure incurred after a certain age (a) is derived by multiplying the dental care expenditure per person by age with the survival rate and added to the total expenses while surviving. The relative lifetime dental expenditure (*RLE_b,a_*) is estimated as 100 × *LE_b,a_/LE_b,0_,* which indicates the percentage of total lifetime expenditures that accrued at or after age a. For example, if *RLE_b,75_ =* 30%, for those with an average life expectancy of 75, medical expenditures to be incurred after age 75 will constitute 30% of the total lifetime medical expenses.

The per capita lifetime expenditure for survivors is an estimate of the lifetime dental expenditure expected to be incurred by a person surviving at a certain age (*x* = *a*). The per capita lifetime expenditure of a survivor at a certain age (a), is given by

$$LE_{s,a}=\overset{95+}{\underset{x=a}{\sum }}CxLx/la$$

where, *Lx* refers to the person-years of survivors in the age group *Lx* = (*χ*, *χ* + 1), *Cχ* refers to dental expenditure per person at age *χ* (*χ* = 0, 1, 2, …, 95 +), and *l*a = (a, a + 1) refers to the number of survivors in each age group. Conceptually, *LE_s,a_* will always be greater than *LE_b,a_*, because the total annual medical expenditure is equal in the numerators while *LE_s,a_* has a lower denominator value.

## 3. Results

### 3.1. Remaining Lifetime per Capita Dental Expenditure for Men and Women, Relative Lifetime Dental Expenditures for Life Table Cohort 

Table 1 presents the gender-based per capita dental expenditure for 2015 by five-year intervals for the age group. The distribution of dental cost for men was as follows: $759, the highest, at age 65–69; followed by $731 at age 35–39; and $668 at age 85–89. On the other hand, the highest spending was $974 for women aged 85–89; the expenditure at ages 55–59 and that over 90 were similar, at $642. 

The study estimated per capita lifetime dental expenditures of the average life table individual in 2015. The average member of the birth cohort at age 0 had an estimated lifetime expenditure of $32,318 for women, and $31,587 for men throughout their life. As per the average life table, women would incur more lifetime expenditure at all ages. The study indicated that the remaining lifetime expenditures were estimated to fall with age as older adults have fewer years remaining to accrue additional expenditure. The per capita lifetime and relative lifetime dental expenditures for both men and women were estimated.

Table 2 shows the relative lifetime dental expenditure. It was the percentage of the total lifetime expenditure that was spent at or after a given age. Men spent about 53% and women about 60% of their lifetime expenditures after the age of 50. Nearly a third of all lifetime expenditure occurred at 65 years and above for men and women. This nearly doubled (21.7%) after the age of 75 in the average life table of women compared to men.

### 3.2. Lifetime per Capita Expenditure by Adjustment for Women’s Longer Life Expectancy

**Figure 2:**
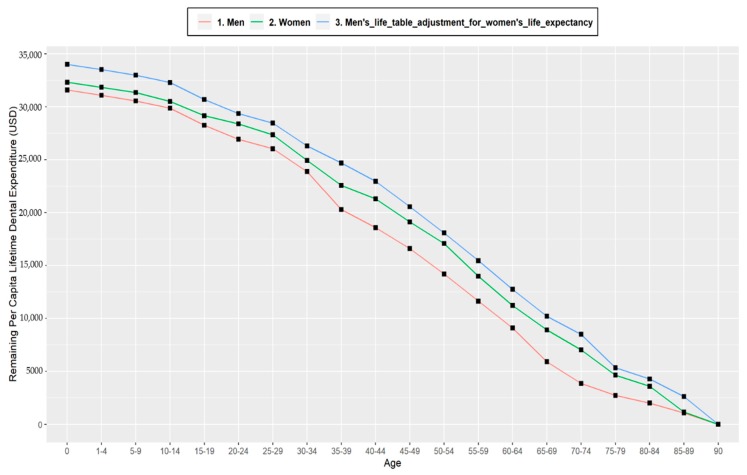
Remaining lifetime per capita dental expenditure by age, men versus women, with/without adjustment for female’s longer life expectancy ($1 USD = ₩1172.5 won in 2015).

Figure 2 presents lifetime per capita expenditure of men, by applying women’s life expectancy to men’s age-specific dental expenditures, attributed to women’s longer lives to determine the increase in expenditure. The men’s life table, unadjusted for women’s life expectancy, would highlight a lifetime dental expenditure of $31,587. If men lived as long as women (adjusted for women’s life expectancy), their lifetime dental expenditure would rise to $34,020. Thus, women’s longer life expectancy impacted nearly 33% of the lifetime dental expenditure difference between men and women. However, additional analysis showed that there was no significant difference between men and women in lifetime expenditure.

### 3.3. Per Capita Lifetime Dental Expenditures for Men and Women, Relative Lifetime Dental Expenditures for Survivors

Table 3 shows the estimated per capita lifetime expenditures among survivors at a given age. The difference in the lifetime expenditure between the survivor and the average life person (Table 1) increased with age because of the diminishing probability of reaching older age. The overall lifetime expenses were higher for surviving men ($38,932) than those for surviving women ($37,772). Surviving women had similar relative lifetime dental expenditures between the survivor and average life person at all ages. In contrast, the difference in the lifetime expenditures between surviving men and average life men increased with age because men were less likely to survive until those ages. At age 65, surviving men had 40.5% of their lifetime dental expenditures ahead of them, compared with 28.9% for the average life person. 

Table 4 displays the per capita lifetime dental expenditures for both the average life table person and survivors at different age intervals. For men, 10.5% lifetime expenditures occurred between 0 and 19 years, 25.2% for young adults (20–39 years), 11.7% for middle-aged people (40–49 years), 23.7% for those aged 50–64, 16.6% for those aged 65–74, and 12.2% for those aged 75 years or older. The pattern was similar for women: 12.9% between 0 and 19 years; 17.3% between 20 and 39 years; 10.7% and 24.4% for women in their middle and old ages, respectively; 13.0% between the ages of 65 and 74, and 21.8% over the age of 75. For a survivor, it showed a pattern similar to the lifetime dental expenditure for the life table cohort, especially that the share of survivor expenditures (24.5%) was more than twice that of the lifetime expenditures (12.3%) for men aged 75 years and older.

## 4. Discussion

This was the first study to simultaneously evaluate the difference in lifetime dental expenditures for both sexes, at various ages for both the average life table person and survivors. The analysis assumed status quo in factors such as healthcare technology and price and the incidence, severity, and outcomes of disease. Results indicated that, by constructing 100,000 virtual cohorts in women, the average expectancy women would spend lifetime dental expenditures of $32,318, which is more than that of the average expectancy of men ($31,587). The results also showed that the average life table women would spend more lifetime expenditures than men throughout their life cycle. The findings supported the claim that the primary factor in which women’s lifetime medical expenses exceeded men’s lifetime medical expenses is the difference in life expectancy [22,23]. 

Figure 2 results indicated substantial differences in lifetime expenditures of both sexes, along with estimations of women’s longevity and their greater lifetime dental expenditures. The findings revealed gender differences in overall dental expenditures, showing women’s expenditures and men’s expenditures in both unadjusted (reflecting men’s shorter life expectancy) and adjusted (if men lived as long as women) cases. The lifetime expenditures were $31,587 for men and $32,318 for women. If men survived as long as women, their dental cost would increase by 33% due to women’s greater longevity. The result presented a clear understanding of the contributions of greater life expectancy to impact dental expenses over a lifetime. Based on these findings, if the upward trend in life expectancy continues, with an increase in the number of older adults due to the aging of baby boomers, the impact on rising dental costs will be significant.

Additionally, the cost of prevention and treatment of oral care paid by patients themselves and through insurers could skyrocket in the future. Korean women have a longer life expectancy than men. Korean men (79.0 years) live 1.1 years more than the Organization for Economic Co-operation and Development (OECD) countries’ average (77.9 years), women (85.2 years) live 1.9 years more than the OECD countries’ average (83.3 years) [26]. A study confirmed that in South Korean women, the probability of living to be over 86 will be 90% by 2030, which was the highest worldwide life expectancy in 2012, and a 57% probability of living for over 90 years [3]. Moreover, the life expectancy of South Korean men was over 80 years, similar to that of Australian and Swiss men. Previous studies confirmed that the aging population contributes to higher dental care expenditures [10,27,28]. The incidence of oral diseases was higher in older adults compared to that in younger people [9,27]. The Korean Health Statistics in 2016 estimated the prevalence of chewing difficulty at about 46.3% for 70 years or older [29]. Previous Korean literature also showed poor oral health, reported by the older adults, for over half of their life cycle [30], suggesting high prevalence and recurrence of caries and periodontitis [8,16]. Therefore, national insurance programs, covering the more expensive treatments (such as dental restoration and prosthetic treatment), are required for Korean older adults. Additionally, the older adults suffered as they had difficulty in gaining access to dental care [31]. Their ability to pay is the most commonly reported barrier in accessing dental care, leading to high oral care spending [28].

A recent study reported that the cost of restoring untreated caries, and the derived benefit (average economic loss) may be incurred in the same year, and every year after that because the incidence of tooth decay can be significant in older people [31]. Thus, it is probable that certain generation changes lead to an increase in overall dental expenditures. The Global Burden of Disease study confirmed that due to demographic changes, including population growth and aging, the cumulative burden of oral disease dramatically increased between 1990 and 2015 [10]. Disease burdens such as dental caries, periodontal disease, and oral cancer increased by 45.6% on average over 20 years, and periodontal disease increased by 58%, showing the highest disease burden [10]. The number of oral diseases (i.e., tooth decay, periodontal disease and oral cancer, trauma) was estimated to be about 3.58 billion worldwide in 2015, including 161 million children with caries and about 700 million people with periodontal disease [10]. These numbers are likely to continue to increase as populations continue growing and aging [10,16]. Thus, it is critical to prevent moderate suboptimal dental conditions starting from earlier stages of life [8], to curb the increase in dental costs. Health investment policies should focus on accessing and using primary and secondary preventive dental services.

Table 2 indicates that analysis is needed to understand the relative lifetime dental expenditures. The study identified that nearly 70% of lifetime costs occur before 65 years of age, and approximately 35% of the lifetime total occurred during middle-age between 40–64 years of age for an average table for men and women. These results showed slightly different patterns of medical expenditure than those presented by Alemayehu and Warner [22] and Spillman and Lubitz [32] who suggested that over half of the lifetime medical expenses occurred after the age of 65. The high incidence of dental expenditure before the age of 65 was attributed to dental trends in Korea. The introduction of Korea’s national dental policies (to cover dental services), which were based on a private fee-for-service plan, was expected to contribute significantly to the increasing dental service utilization. For example, the Korean government implemented an initiative to achieve universal healthcare coverage including the dental sealant for children and adolescents (since 2009) as well as the cost of dental scaling in 2013, resulting in higher cost-sharing (70–90%) and lower out-of-pocket expenses (30–10%), a move to improve the level of oral health [33,34]. These progressive policies positively improved accessibility by eliminating barriers to access to dental care, namely, increased total outpatient cost for dental services in recent years [35,36]. Certain changes in dental policy probably lead to an increase in overall dental expenditures before the age of 65 during the life cycle.

The analysis of survivors’ lifetime costs showed a similar proportion for an average living person before the age of 65 (Table 3). Interestingly, the proportion of lifetime dental expenditures among age groups was not uniform (Table 4). These results are important because analysis of age-specific increased spending is essential to predict dental costs as society ages, given the disproportionate healthcare resources supporting older adults. Thus, it is crucial to plan for future needs to increase the dental care resources supporting older adults throughout their life cycle. Ensuring access to dental care for prevention may partially reduce the burden of dental conditions over life [8,37]. Policymakers ought to address the interaction of age with dental care spending in the rapidly aging population and consider effective national strategies for the inclusion of dental care coverage in health-insurance packages. Although the study is based on data from 2015 and relates to lifetime costs, it would be important to apply age-specific estimates to the age distribution to examine patterns of dental expenditure resulting from population aging.

This study had several limitations: First, identifying lifetime dental expenditures and treatment costs for orthodontic or aesthetic purposes, incurred by children and young adults (most patients pay in full for non-covered items under the health insurance system). The expected lifetime dental expenditures may be lowered by excluding the costs that are not directly related to oral healthcare. Second, information on the costs associated with death could not be provided due to the limitations of the data. Previous studies on estimated lifetime medical expenditures have highlighted that the difference in medical costs between deceased persons and survivors, at a specific age, was difficult to explain. These estimates could be lowered as medical costs for people who die at a certain age could be more significant than that for those who normally survive [21,22]. For example, the difference in medical costs between deceased persons and survivors at a specific age may be lowered as costs associated with chronic illnesses may increase as age increases. Still, the medical costs of people who die at a certain age may be consistently higher than that of survivors. Nevertheless, the study found no evidence that dental care expenses were incurred at the time of death in the life cycle, making it unlikely that our total lifetime medical expenditures were underestimated. Third, health behaviors, such as smoking and alcohol drinking and other factors such as medical and dental history, can affect the difference in medical costs by gender. Future research will require extensive consideration of a range of factors that may distort the difference in medical expenditure. Fourth, the cross-sectional data and life tables used in this study only estimate the medical expenditure for the current year. Therefore, the hypothetical cohort’s survival rates and changes in dental care expenses, at various points in time, could not be reflected. Consequently, it would be necessary to continuously monitor the trends based on changes in life expectancy in Korea.

## 5. Conclusions 

This study estimated the lifetime dental expenditures of both men and women, throughout their life, using the life table. This study found that a Korean spends over $30,000 through their lifetime. The average lifetime expenditure for men was about 33% greater than the estimated women’s lifetime expenditure if they survived as long as women. The study highlighted the need for a comprehensive public policy in the future as the society ages, given the disproportionate age-specific dental spending throughout a Korean’s life cycle.

## Figures and Tables

**Table 1:** Per capita death rate, dental expenditure, and lifetime dental expenditures of average life table men and women.

Age	Death Rate		Dental Expenditure per Capita		Lifetime Dental Expenditure	
	Men	Women	Men	Women	Men	Women
0	0.00292	0.00251	-	-	$31,587	$32,318
1–4	0.00073	0.00055	$124.86	$118.30	$31,089	$31,846
5–9	0.00051	0.00035	$106.73	$98.23	$30,557	$31,356
10–14	0.00053	0.00032	$138.07	$171.24	$29,870	$30,504
15–19	0.00130	0.00075	$323.92	$471.36	$28,259	$29,156
20–24	0.00214	0.00112	$266.21	$355.53	$26,937	$28,388
25–29	0.00298	0.00168	$182.31	$409.08	$26,034	$27,355
30–34	0.00365	0.00241	$435.88	$425.83	$23,883	$24,931
35–39	0.00504	0.00300	$731.21	$275.73	$20,289	$22,568
40–44	0.00826	0.00413	$349.67	$257.50	$18,581	$21,299
45–49	0.01384	0.00580	$409.36	$445.08	$16,604	$19,117
50–54	0.02156	0.00766	$507.69	$417.66	$14,194	$17,083
55–59	0.03097	0.01078	$543.84	$642.80	$11,630	$13,981
60–64	0.04578	0.01627	$568.39	$578.40	$9102	$11,226
65–69	0.06852	0.02677	$759.56	$495.49	$5914	$8916
70–74	0.11724	0.05289	$537.49	$419.32	$3859	$7035
75–79	0.20762	0.10752	$350.95	$574.93	$2730	$4655
80–84	0.33656	0.21343	$300.69	$302.55	$2020	$3595
85–89	0.50259	0.38194	$668.40	$974.11	$1091	$1172
90–	1.00000	1.00000	$144.35	$642.99	-	-

Note: The death rate was calculated from the abridged life table (five-year intervals for the age group) for 2015 from the South Korean Statistical Information Service (KOSIS). Dental expenditure per capita by age was calculated from the Korea Health Panel Survey (2015).

**Table 2:** Per capita lifetime dental expenditures of average life table and the relative lifetime dental expenditures for men and women.

Age	Men		Women	
	Lifetime per Capita Dental Expenditure	Relative Lifetime Dental Expenditure	Lifetime per Capita Dental Expenditure	Relative Lifetime Dental Expenditure
0	$31,587	100%	$32,318	100%
20	$26,937	89.5%	$28,388	87.1%
40	$18,581	64.3%	$21,299	69.8%
50	$14,194	52.6%	$17,083	59.1%
65	$5914	28.9%	$8916	34.7%
75	$2730	12.3%	$4655	21.7%

$1 USD = ₩1172.5 won in 2015.

**Table 3:** Per capita lifetime dental expenditures of average life table and the relative lifetime dental expenditures for survivor men and women.

Age	Men		Women	
	Lifetime per Capita Dental Expenditure	Relative Lifetime Dental Expenditure	Lifetime per Capita Dental Expenditure	Relative Lifetime Dental Expenditure
0	$38,932	100%	$37,772	100%
20	$34,261	91.4%	$31,819	88.9%
40	$25,786	70.7%	$24,987	65.9%
50	$21,240	61.0%	$20,687	60.2%
65	$12,104	40.5%	$12,172	38.6%
75	$7978	24.5%	$7389	26.7%

$1 USD = ₩1172.5 won in 2015.

**Table 4:** Lifetime dental expenditure for life table cohort and survivor men and women by life cycle.

Age Intervals	Life Table Cohort		Survivors	
	Men	Women	Men	Women
0–19	10.5%	12.9%	8.6%	11.1%
20–39	25.2%	17.3%	20.7%	23.0%
40–49	11.7%	10.7%	9.7%	5.7%
50–64	23.7%	24.4%	20.5%	21.6%
65–74	16.6%	13.0%	16.0%	11.9%
75	12.3%	21.8%	24.5%	26.7%
